# Temporal dynamics of microbiota before and after host death

**DOI:** 10.1038/s41396-018-0157-2

**Published:** 2018-06-04

**Authors:** David Preiswerk, Jean-Claude Walser, Dieter Ebert

**Affiliations:** 1 Department of Environmental Sciences, Zoology, University of Basel, Vesalgasse 1, Basel, CH-4051 Switzerland; 2 Genetic Diversity Centre (GDC), Universitätstrasse 16, Zürich, CH-8092 Switzerland

## Abstract

The habitats that animals, humans and plants provide for microbial communities are inevitably transient, changing drastically when these hosts die. Because microbes associated with living hosts are ensured prime access to the deceased host’s organic matter, it is feasible that opportunistic, adaptable lifestyles are widespread among host-associated microbes. Here we investigate the temporal dynamics of microbiota by starving to death a host—the planktonic Crustacean *Daphnia magna*—and tracking the changes in its microbial community as it approaches death, dies and decomposes. Along with obligate host-associated microbes that vanished after the host’s death and decomposers that appeared after the host’s death, we also detected microbes with opportunistic lifestyles, seemingly capable of exploiting the host even before its death. We suggest that the period around host death plays an important role for host–microbiota ecology and for the evolution of hosts and their microbes.

## Introduction

Multicellular eukaryotes provide habitats for symbiotic microbial communities on their body surface and in its cavities [1, 2]. These habitats are transient, however, lasting only as long as the host’s lifespan; when the host dies, its microbes suddenly find themselves in dead organic matter [3]. Symbiotic microbes must, thus, either replicate and disperse to new, living hosts before host death, or adjust to this change in habitat quality. Previous studies of host-associated microbiota have usually focused on host fitness and ecology, exploring the composition and dynamics of microbiota in relation to the living host [1, 4–6]; however, this focus is relevant primarily for obligately host-associated microbes. On the other end of the spectrum are saprotrophic microbes, which colonize the host’s carcass from the environment after it has died [7–10]. These microbes have been well explored in ecology and forensic science studies that track the changing composition of the microbial community in decomposing animals [11–14]. However, microbes capable of a saprotrophic lifestyle may already be present in the microbiota of the living host. These “opportunists” may persist in the living host and flourish in the dead host as abundant resources become available [15–17], thereby gaining a temporal and numerical advantage over saprotrophes (“first come, first served”). This opportunistic strategy has not been well explored, as most studies in the field of carrion ecology follow the dynamics of microbial communities only after host death (reviewed in ref. [3]). Moreover, these opportunists may not only benefit from sudden host death, but may reap their full advantage if host death is predictable, as, for example, at the end point of a disease, or starvation, or in an aging host. In these cases, opportunistic microbes may take advantage of the dying host’s declining vital functions, such as impaired immune response and low resource levels [18]. To gain a deeper understanding of the functional ecology of microbes and the range of microbe lifestyles, this study examines the post-mortem fate of microbes already present in the living host, using established concepts in carcass ecology, where microbial dynamics in decomposing carcasses follow a deterministic time course [11–14].

**Fig. 1:**
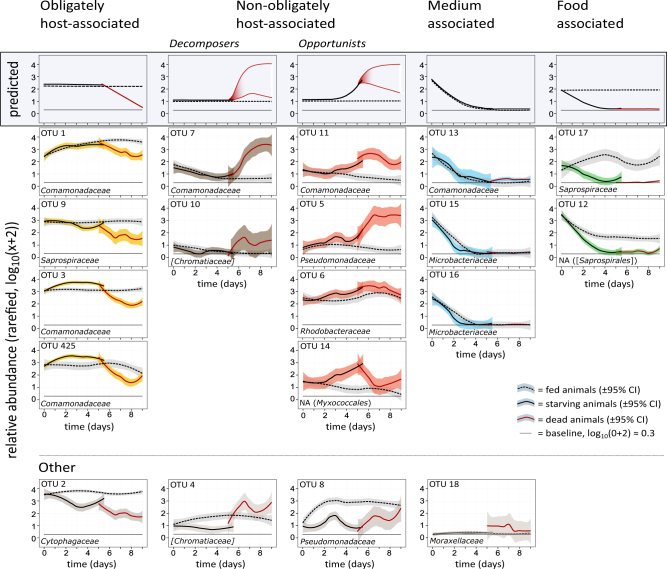
Classification of OTU dynamics into five groups, with each shape indicating a particular lifestyle in relation to the fed, starving and dead host. The predicted lifestyle patterns are given at the top. The relative abundance of the OTUs over time in the fed, the starving and the decomposing animals were approximated with the Loess function in R. The 95% confidence intervals are drawn around the lines. OTU numbers are given above the graphs, and bacterial families below (in square brackets if candidate taxon). Confidence intervals of the OTU-15, OTU-16, and OTU-17 are very narrow for the dead animals and, hence, barely visible. Time 0 (t0) represents the samples taken before treatments were applied. The first point in the decomposition of the dead animals is day 5, the upper boundary of the 95% CI interval of the median time of death

As a model for this study, we used the planktonic crustacean *Daphnia magna*, which typically harbors and depends upon a few dozen microbes [19–21] for growth, reproduction and survival [22]. We tracked the composition of the *Daphnia*’s microbiota as the host starved, died and decomposed. By inducing starvation as a non-invasive cause of death, as opposed to a sudden death, we were able to observe whether there were opportunistic microbes in the system that took advantage of their host (as indicated by an increase in abundance) during the time when the host’s vital functions were declining. Death by starvation is common in the natural ecology of *Daphnia* and its microbiota, as food resources become overexploited [23–26]. Our experiment compared starved animals with well-fed animals, and restricted the arrival of environmental microbes after the starvation treatment had begun. Our hypothesis was that the changes in microbial abundance around the time of host death would follow diverse patterns (Fig. 1, top row), the more prominent once are: (1) an obligately host-associated lifestyle, where the relative abundance declines at the point of host death; (2) a saprotrophic lifestyle, where the relative abundance increases after host death (decomposer lifestyle), and (3) an opportunistic lifestyle, where relative abundance begins increasing already before the host’s death and persists for some time after host death. We also expected other patterns to arise because of our specific experimental conditions: (4) Medium-associated microbes, which decrease in abundance the moment all animals are placed in microbe-free medium and the arrival of environmental microbes is restricted (before this, animals were kept in non-sterile medium in an open system); and (5) food-associated microbes, which are associated with the host’s food or its feeding physiology and decrease in abundance and disappear in starving animals, but persist in fed animals.

## Materials and methods

### Starvation experiment

*Daphnia magna* were sampled in a eutrophic pond (about 200 × 300 m surface area, maximum depth 3.8 m; N 47.558°, E 8.862°) [27] and brought to the laboratory where individual females were cloned (iso-female lines). Clone CH-H-149 was arbitrarily chosen for this study. A cohort of females from clone CH-H-149 were raised in the lab in 120-mL jars with ADaM medium [28] at a density of three animals per jar. All animals were fed on an exclusive diet of chemostat grown green algae (*Scenedesmus obliquus*) [29]. Offspring were removed daily. After 3 weeks, 60 randomly chosen females with well-visible clutches in their brood pouch were brought together in one 1.5-L jar and fed with algae ad libitum. After 24 h, the mothers were removed from the jar, and all offspring (born within 24 h) were kept together for the following four days and fed with algae ad libitum. Until this time, the food and medium were taken from open resources in the laboratory and had not been sterilized [30, 31]. When the animals were four days old, we transferred 308 of them to individual jars with sterile, filtered (0.2 μm) ADaM and assigned them randomly to either the feeding or the starvation treatment (154 animals each). Before the assignment of treatments (t0), three four-day-old animals were sampled and stored in 2-mL tubes at −20 °C. From this point on, only sterile, filtered ADaM was used. Animals in the feeding treatment were fed 5 × 10^6^ cells of algae daily from one single batch of algae that had been frozen in aliquots. All animals were transferred to fresh jars with sterile ADaM daily. Jars were covered with fresh cling film after every handling and kept in an incubator with a 16/8 h light/dark cycle at 20 °C. Every 12 h, we randomly chose five animals in the feeding and five animals of the starvation treatment, transferred them with one drop of medium into individual 2-mL tubes and froze them at −20 °C. Animals that died in the starvation treatment were assigned, on a rotating basis, to a future time point, post-death, when they would be sampled—9 sampling times at 12 h intervals between 0 and 96 h after death. In this way, we obtained eight groups of carcasses to sample at different time points in the decomposition process that corresponded with the intervals as the live fed and starved animals. Sampling and treatment of the fed animals were continued until the last dead animal from the starvation treatment was sampled (see Figure S1 in supporting information). The analysis included the three samples taken at t0 before treatments were assigned and three randomly chosen replicates per time point and treatment (at least two further samples per time point had been collected as a back-up, but were not used). However, for the fed animals, we included only every second time point (24-h intervals), as we expected less change and wanted to achieve an approximately equal number of samples across the three sampling groups. In total, we analyzed three animals from the pre-treatment phase, 27 fed animals (9 time points across 9 days), 33 starved, living animals (11 time points in 12 h intervals) and 27 dead animals (9 time points in 12 h intervals with). Each sample was destructive, i.e. we used the entire animal or carcass, resulting in totally independent replicates. The medium and the food were not sampled.

### Next-generation amplicon sequencing

The molecular work was done in collaboration with Genetic Diversity Centre in Zürich (GDC). Samples were randomly assigned to batches for extraction and library preparation. DNA was extracted from the samples using the CTAB method [32] and quantified using a high sensitivity dsDNA assay on the Qubit fluorometer (Q32857). DNA concentration was then normalized to ≤0.5 ng/µL. Mock samples (artificial communities, i.e., positive controls produced from cultured bacteria) and negative controls for the DNA extraction and PCR were included in all downstream steps.

A two-step PCR approach was used to amplify a ca. 440 nt region of the 16 S rDNA gene covering variable region V3–V4 in prokaryotes (excluding eukaryotes) and adding adaptors for sequencing on an Illumina MiSeq platform. The primers of the first PCR were composed of the target region and an Illumina Nextera XT specific adapter sequence. Four sets of forward and reverse primers, which differ by 0–3 additional random bases between primer and adapter sequence, were mixed. These additional bases introduce frameshifts into the sequencing process to increase complexity. The primers were used in equimolar combinations. For each sample, a 25-µL PCR reaction was performed with the following specifications: 2 µL sample, 23 µL H_2_O, 0.75 µL forward-primer mix, 0.75 µL revers-primer mix (R), 0.5 µL dNTP (ThermoScientific), 0.25 µL Polymerase and 5 µL Buffer A, both from the KAPA2G Robust hotstart kit (KAPA Biosystems®), cycling program: 1 × 95 °C 5 min, then 22 × (95 °C 30 s, 58 °C 15 s, 72 °C 30 s), then 1 × 72 °C 3 min, finally hold at 4 °C.

The amplified DNA was purified using Ampure XP beads from Beckman Coulter according to the manufacturer’s protocol. Subsequently, the adapter tagged libraries were individually indexed in a 10-cycle indexing PCR using 2xKAPA HiFi hotstart readymix (KAPA Biosystems) and Nextera XT V2 kit (Set A and Set D, Illumina, San Diego, USA). PCR reaction (50-µL): 25 µL 2 × KAPA readymix, 5 µL Nextera XT index (N7xx), 5 µL Nextera XT index (S5xx), 15 µL sample (purified PCR product), cycling program: 1 × 95 °C 3 min, then 10 × 95 °C 30 s, 55 °C 30 s, 72 °C 30 s, then 1 × 72 °C 5 min, finally hold at 4 °C. The indexed libraries were purified again using Ampure XP beads. The concentration of each library was determined through qPCR using KAPA Library Quantification Kit (KAPA Biosystems) according to the manufacturer’s protocol. DNA concentrations of the indexed libraries were normalized. The pooled libraries were again purified using Ampure XP. The fragment distribution of the pooled libraries was then determined using an Agilent Bioanalyzer, and the final concentration of the pooled libraries was determined using qPCR. The pooled libraries were denatured and spiked with a PhiX library according to the manufacturer’s protocol. The libraries were then loaded to the MiSeq cartridge and sequenced in a 300-bp paired-end run on Illumina MiSeq.

Raw reads were quality controlled [33] (FastQC: a quality control tool for high throughput sequence data. Available online at: http://www.bioinformatics.babraham.ac.uk/projects/fastqc); paired reads were merged (FLASH v.1.2.9, minimum overlap: 15, max overlap: 250, max mismatch density: 0.25 [34]; primers were trimmed from the merged reads (cutadapt v.1.5, overlap: full length, error rate: 0.01, wildcards allowed [35]); and trimmed reads were quality filtered (PRINSEQ-lite v0.20.4, fragment length: 350–550, GC range: 30–70, minimum quality mean: 30, no ambiguous nucleotides [36]). UPARSE (usearch v7.0.1090_ i86linux64 [37]) was used to remove chimera and map operational taxonomic units (OTUs, 97% sequence identity). OTUs represented by a single read (abundance below 1) were removed from the table. Taxonomic assignment was done through a blastn search against the GreenGenes database (13_5, http://greengenes.lbl.gov/), and reference sequences were assigned to the OTUs.

Sequences were aligned using PyNAST [38] with a minimal sequence identity threshold of 55%. A phylogenetic tree was produced with FastTree [39], which infers approximately-maximum-likelihood phylogenetic trees from alignments of nucleotide sequences. PyNAST and FastTree are part of Qiime (v 1.8.0) [40].

### Statistics

Data analysis was done using R [41]. Rarefaction, diversity estimates and double principal coordinate analysis (DPCoA) [42] were done using the R package Phyloseq [43]. Linear regression analysis of Simpson’s index over time was done using the raw OTU counts per sample before rarefaction. Samples were rarefied to the sample with the least reads. OTUs present over multiple points in time were selected by two criteria: they had to be present in at least six samples and had to represent over 0.5% of the remaining reads. Community composition was then analyzed using ADONIS based on DPCoA distances (R package vegan) [44]. For this analysis, we excluded the t0 samples that were taken before treatments were assigned. Temporal dynamics of relative abundance (log_10_ transformed (log_10_(x + 2)) were approximated by local polynomial regression (loess; geom smooth function, R package ggplot2 [45]) with time as the continuous independent variable. Survival and the median time of death were analyzed using the R package “survival” [46].

## Results

### Starvation and Death

In the starvation treatment, we sampled 53 live animals and 99 animals that died from starvation between 3.5 and 5.5 days (median time to death = 4.5 days; 95% CI: 4.5 to 5 days). The dead animals were assigned to nine groups over time post death (see Methods for details) to track microbiota over different stages of decomposition. Of the fed animals, only two of 154 died during the experiment; those animals were excluded from sampling. Overall, the analysis included three animals from before the treatments were assigned (t0), 27 fed animals, 33 starved animals, and 27 animals that had died from starvation (total *n* = 90, 3 per treatment × time combination).

### Temporal patterns in relative microbe abundance

**Fig. 2:**
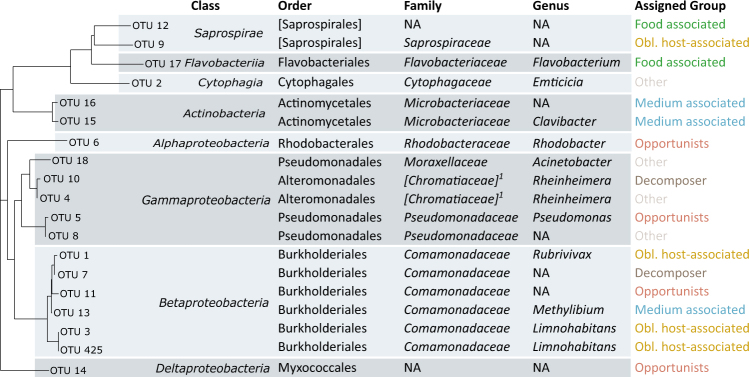
Phylogenetic tree and full taxonomic assignment of the most abundant OTUs. Taxonomic groups given in square brackets represent candidate classes

Next generation sequencing yielded 22.7×10^6^ reads, of which 12,763,890 passed the quality filtering. Analysis of mock samples (positive controls) consistently revealed the expected bacteria, and negative controls revealed no systematic contamination. We obtained 556 taxonomically assigned OTUs at 97% identity (without controls), all of which were assigned to the bacteria. Samples were rarefied to 15,390 reads each. After rarefaction and removal of OTUs that did not pass our abundance criteria, 19 OTUs remained in the dataset (belonging to eight bacterial classes, Fig. 2), representing 97% of the rarefied reads before the abundance criteria filter had been applied.

None of the 19 common OTUs observed in our study belonged to the Firmicutes (Fig. 2), a phylum commonly observed in living vertebrates and their decomposing carcasses [13, 47]. Furthermore, Actinobacteria, also common in decomposing vertebrates [13, 47] were found only in association with the medium used in our study (Fig. 2). We did, however, find Gammaproteobacteria, also common in vertebrates.

Temporal dynamics of the individual OTUs were approximated by local polynomial regression (loess) for the fed, starving and dead animals (Fig. 1). The starting point (t0) represents the samples before treatments were applied. In Fig. 1, the first data for the dead animals begins at day 5, the upper boundary of the confidence interval of the median time of death. The temporal dynamics of relative abundance for the 19 OTUs followed diverse patterns, leading us to tentatively assign them to one of the following groups: (1) Obligately host-associated, (2) Non-obligately host-associated/Decomposers, (3) Non-obligately host-associated/opportunists, (4) Medium-associated, (5) Food-associated, and (6) Other (Fig. 1). Although this categorization is not absolute and is subject to debate, it illustrates the marked diversity of the OTUs and highlights that their dynamics can provide functional clues about their different lifestyles. The assignment of OTU dynamics to these groups, thus, enables a structured discussion of the observed patterns, although it must be treated as hypotheses.

### Obligately host-associated microbes

Four OTUs were placed into the category of obligately host-associated microbes. All of them show a rapid decline the moment the host died, suggesting that they depend on a living host. OTU-1, -3 and -425 are members of the *Comamonadaceae* family (Fig. 2), with OTU-3 and -425 belonging to the genus *Limnohabitans*, which colonizes the filter apparatus of *Daphnia*, where dissolved organic nutrients are taken up from the water [48]. OTU-9 belongs to the *Saprospiraceae* family in the phylum *Bacteroidetes*.

### Non-obligately host-associated microbes

The six OTUs we assigned to the non-obligately host-associated microbes showed an increase in relative abundance shortly before (opportunistic life-style) or after (decomposer) host death (Fig. 1). The opportunists OTU-11 and OTU-5 increased in abundance about 1 to 1.5 days before the median time of host death. OTU-5 is a member of the *Pseudomonadaceae*, which contains several opportunistic pathogens [49]. OTU-11 is a member of the *Comamonadaceae*, a diverse family with many members belonging to the *Daphnia* microbiota [20]. OTU-6 (genus *Rhodobacter*) increased slowly before host death, maintained its abundance, and then declined again after 2.5 days. As *Rhodobacter* have phototrophic capabilities [50], OTU-6 may be somewhat independent from heterotrophic metabolism. OTU-14 (*Myxococcales*) decreased slowly in the fed animals over time, but increased substantially in the starving animals from day two until the host’s death, at which point the relative abundance started to decline again.

In both the starving and the fed animals, the two OTU (7 and 10) classified here as decomposers exhibited a marked increase in relative abundances after host death. OTU-7 belongs to the *Comamonadaceae*, which have been associated with decomposition under hypoxic conditions [51]. OTU 10 belongs to the candidate-family *Chromatiaceae*, which can maintain a photoautotrophic lifestyle under anoxic conditions and is thought to play a role in decomposition under anoxic conditions [52].

### Medium- and food-associated microbes

OTU-13 (*Comamonadaceae*), OTU-15 and OTU-16 (both *Microbacteriaceae*) are classified here as medium-associated because they disappeared in both the fed and the starving animals within two to three days after we switched the animals to sterile, filtered medium (Fig.1). The decrease may be a dilution effect caused by the daily transfers of the animals to fresh, sterile medium, but *Daphnia* might also have feed on them. Both the genus *Methylibium* (OTU 13) and diverse members of the family *Microbacteriaceae* (OTU-15, OTU-16) have been found in our culture medium in other projects (unpublished data, Samuel Pichon & Dieter Ebert).

OTU-12 (*Saprospirales, Bacteroidetes*) and OTU-17 (*Flavobacteriaceae, Bacteroidetes*) persisted in the fed animals over the entire period of the experiment but decreased rapidly in the starving animals (Fig.1). These bacteria are either part of the food, or they grow in *Daphnia* as long as the host is feeding on this food.

### Other dynamics

A poor match between the predicted and the observed pattern (group “other”, Fig. 1) can occur for various reasons: the biology of an OTU may not follow any of the predicted patterns, or an observed pattern may be distorted by statistical noise in the data, or there may be potential biases in methods. Potential biases include the sequencing method (short reads, sequencing errors), issues with species delineation (OTUs may not represent single species, but may include two or more species with high sequence similarity), and the use of relative abundances. As the dynamics of the “other” group (Fig.1) cannot be definitively explained, or predicted, we abstain from making undue speculations here.

### Community composition and diversity

**Fig. 3:**
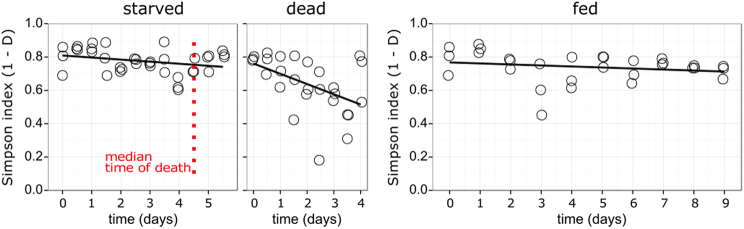
Linear regressions of Simpson’s diversity index (1-D) over time. The decrease in alpha diversity was marginally significant during starvation (R^2^_Adj._ = 0.062, *p* = 0.08) and significant after host death (R^2^_Adj._ = 0.2, *p* = 0.01). Alpha diversity did not change in the fed animals (R^2^_Adj._ = 0.007, *p* = 0.28). To calculate diversity, the dataset with all OTUs before rarefaction was used

**Fig. 4:**
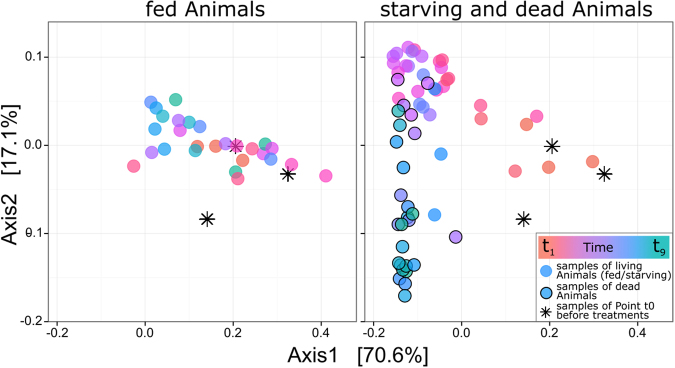
DPCoA plots (Axis 1 and 2 with % variance explained) of the samples separated by treatment. The dead, decomposing samples of the starvation treatment are shown as circles with a black edge. Asterisks indicate the t0 samples from before treatments were applied. Time is represented as a color gradient. The fed animals form a stretched cluster, exhibiting relatively little change in community composition over time. The starving samples co-localized with the fed samples only at the onset of the experiment (orange). With ongoing starvation, the communities changed continuously and moved to the upper left corner (red-purple), then shifted downwards along axis 2 when the animals were approaching death (blue-green)

The alpha diversity of the microbiota stayed the same in both the starved and the fed animals. Only during the decomposition of the dead hosts did it decrease (Fig. 3). Using Double Principal Coordinate Analysis (DPCoA) [42], we graphed the dynamics in community composition over time. By plotting all samples along the first two axes of the DPCoA (explaining 87% of the variability), we saw a substantially larger variance in community composition in the starving and dead samples than in the fed samples (Fig. 4). When we represented time as a color gradient, a continuous development of community composition during starvation, towards death and then through decomposition became visible (ADONIS analysis: Treatment: *R*^2^ = 0.54, *p* = 0.0002; time: *R*^2^ = 0.03, *p* = 0.003; treatment x time interaction: *R*^2^ = 0.05, *p* = 0.002; supporting information S2). Running an ADONIS on the three groups separately confirmed the effect of time on community composition through starvation (*R*^2^ = 0.34, *p* = 0.0004) and after death (*R*^2^ = 0.16, *p* = 0.006), but not in the fed animals (*R*^2^ = 0.09, *p* = 0.11; supporting information S2).

## Discussion

The ecological significance of microbes obligately associated with living hosts and saprophytic microbes associated with decomposing organic matter are well known [2, 53, 54]. The transformation of a living host to dead organic matter at host death inevitably connects these two realms. Here, we investigated the dynamics in the relative abundance of the microbiota across the entire time period during which a host approaches death, dies and decomposes. Despite the extreme heterogeneity of microbial dynamics, the alpha diversity of the communities did not differ between starving and fed animals, but it did decline after the onset of decomposition, emphasizing host death as an incisive habitat transition [13, 55]. The persistence and dynamics of various OTUs beyond host death, however, show that host death is not equally incisive for every member of the microbiota.

Consistent with earlier studies [13, 47], our analysis shows that the relative abundance of common members of the microbiota ranges widely, displaying various temporal dynamics that testify to the existence of different microbial lifestyles. Indeed, forensic science uses these diverse dynamics to estimate the time since host death [12, 56], although this method is typically not applied to invertebrates. Interestingly, we detected strongly divergent lifestyles among the microbiota, even within the same bacterial family (Fig. 2). Two of the microbial lifestyles we predicted and observed here—obligate host-associated microbes, which grow in living hosts versus decomposers, which grow in dead organic matter—are often-used classifications for microbiota in ecology. However, in accordance with our hypothesis, our study also found microbes belonging to the decomposers that already lived, in appreciable abundance, in living hosts. These OTUs did not conform to the stereotypical picture of a decomposer or an obligate host-associated microbe, but instead seem to be opportunistic microbes that increase even as the host approaches death and continue as saprophytes for some time after it dies. To our knowledge, such opportunistic microbes have not been described before, although some studies have shown that carcasses with restricted access to environmental microbes (as in our study) do decompose more or less normally, albeit more slowly, than carcasses invaded by microbes from the gravesoil and to have a different microbiota composition [9, 57]. These studies, however, did not examine the dynamics of the microbiota before host death.

The observation that both opportunists and the decomposers are present in living, healthy hosts (including the fed samples) indicates that these microbes may adopt a kind of sit-and-wait (SAW) strategy. This strategy could be adaptive: the former free-living decomposers profit by colonizing a living host and waiting in an inactive stage until the host dies [58], thereby gaining a competitive time advantage over environmental microbes that have remained free-living and colonize the host only after its death. As opposed to traditional SAW predators, SAW decomposers and opportunists have a guaranteed chance of success, as every host eventually dies. This strategy may represent an initial step in the evolution of opportunistic lifestyles and may set the stage for the evolution of pathogens that also thrive as saprophytes [59, 60].

Finally, our discussion of these data is based on the classical labeling of pairwise interactions between species, with terms referring to the costs and benefits between two partners, such as commensal, mutualist and parasite. It has recently been argued that such labels are not meaningful if the interaction of two specific partners is not a dominant feature of the entire system, as is usually the case in microbiota–host interactions where multiple microbe species interact with each other, the host, and with the environment [61, 62]. This study contributes to that discussion by pointing out that it is difficult to assign many of these microbes to clear categories. Moreover, while it is possible to group microbes into clusters based on some features, this clustering depends to some degree on what categorizing features one chooses. Because most of our reasoning was based on a pairwise interaction between the host and individual microbe species, we did not include the possibility that some of the observed dynamics might be dominated by interaction between two or more microbes and their response to the changing environment. Thus, our classifications in Fig. 1 need to be seen with this caveat. Even though the graphical model seems to provide neat categorizations for several OTUs, these classifications were most easily applied to the environmental microbes (medium and food-associated, Fig. 1), which have less complex biological interactions with other members of the system and could be clearly distinguished in our analysis from microbes that have an association with the host. In earlier *Daphnia* microbiota assessments, such environmental microbes were considered part of the *Daphnia* associated microbiota [20, 63]. For other microbes, our graphical model and classification provide a working hypothesis for further investigations.

## Conclusion

Our findings point to many exciting questions in both microbial ecology and in the evolution and ecology of symbioses along the multiple dimensions of mutualistic, parasitic and saprophytic lifestyles. Since the organic matter available after host death is built up during the host’s lifetime, it brings together host fitness, host–microbiota interaction and microbiota ecology, extending the relationship between host and microbiota beyond host death, to include decomposition as the last stage of this symbiosis. Members of the microbiota that are useful in some way to the host but do not gain any fitness from this association during the host’s lifetime might ultimately profit after host death. The proliferation of certain microbes after the host’s death also contributes to the microbiome of the surrounding environment, adding to ecosystem function and local microbial diversity [14, 64]. Our research may also relate to the role of microbiota in aging hosts, a relatively unexplored field [18].

As our data show, the lifestyles of certain members of the microbiota cannot be easily classified into traditional categories such as saprophyte or mutualist, but rather fall along a spectrum ranging from symbionts (pathogenic or mutualistic) to saprophytes. Although pathogenic symbionts that also act as saprophytes have previously been described [59, 60], it is unclear whether there are mutualists out there that continue living as saprophytes once their host dies. Our study did show, however, that a fair number of common microbes seem able to survive in both a living host as well as its decomposing remains, suggesting that the capability to switch opportunistically between a host-associated and a saprophytic lifestyle may be common.

### Data accessibility

The sequencing data generated in this study have been deposited in the public European Nucleotide Archive (ENA) server. The accession number for the study is PRJEB26643, the accession numbers for the samples are ERS2473695–ERS2473793.

## Electronic supplementary material

SUPPLEMENTAL MATERIAL 1

SUPPLEMENTAL MATERIAL 2
