# Supplementary figures and images for: Temporal dynamics of microbiota before and after host death
Source: ISME J. 2018 Jun 4;12(8):2076–85. doi: 10.1038/s41396-018-0157-2 (PMC6052066; doi:10.1038/s41396-018-0157-2)

Appendix S1: Sampling scheme

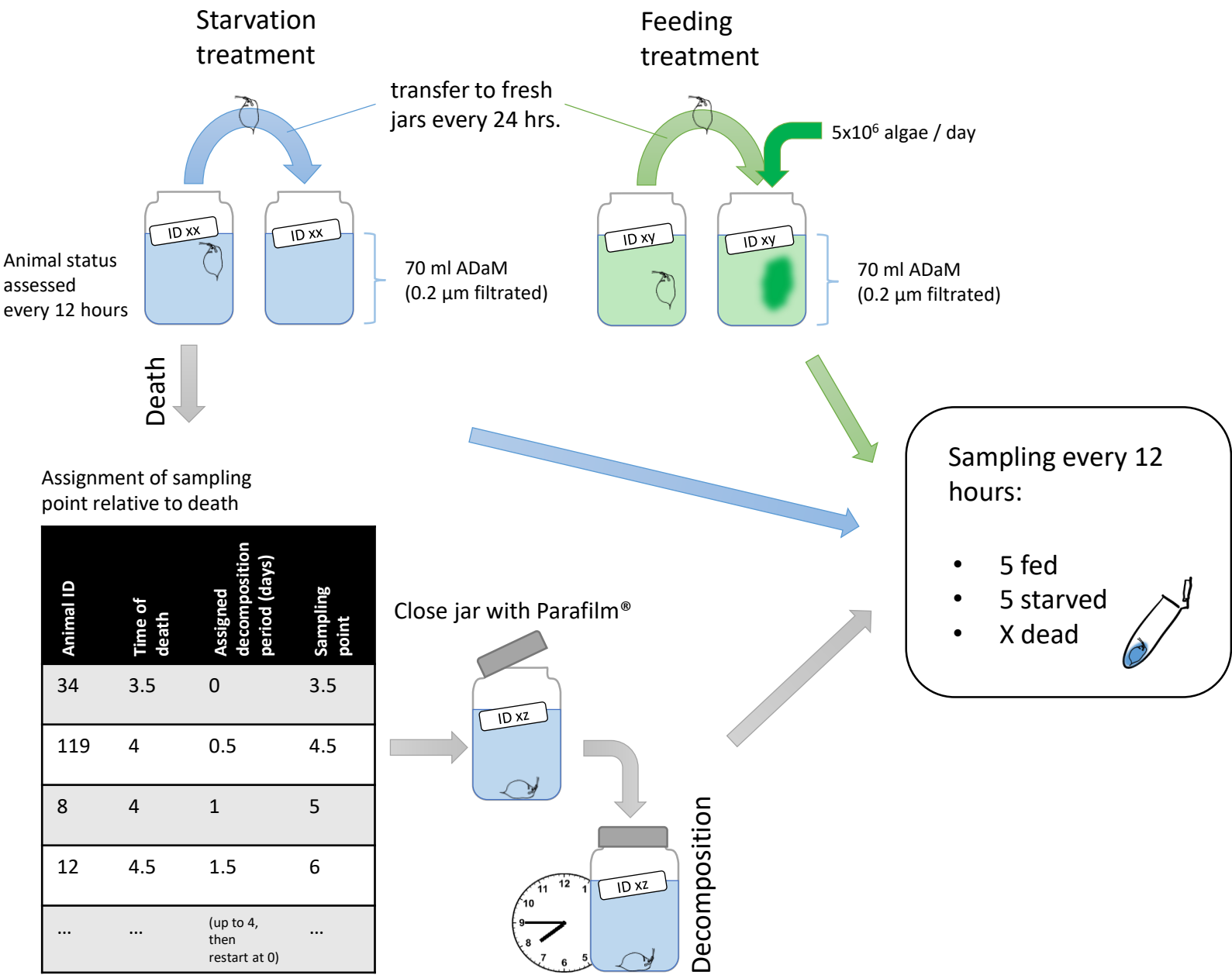

Supplement: Supplementary file 1 — SUPPLEMENTAL MATERIAL 1 [file 41396_2018_157_MOESM1_ESM.pdf]
