# Supplementary material for: Temporal dynamics of microbiota before and after host death
Source: ISME J. 2018 Jun 4;12(8):2076–85. doi: 10.1038/s41396-018-0157-2 (PMC6052066; doi:10.1038/s41396-018-0157-2)
Supplement: Supplementary file 2 — SUPPLEMENTAL MATERIAL 2 [file 41396_2018_157_MOESM2_ESM.pdf]

Appendix S2: Results of ADONIS analysis of DPCoA distances

ADONIS of all Samples / Treatments combined:

	Df	*SS*	*F*	*R2*	*Pr(>F)*	
time	1	0.08527	7.024	0.03294	0.002999	**
treatment	2	1.38529	57.051	0.53517	0.0002	***
time:treatment	2	0.13454	5.541	0.05197	0.0018	**
Residuals	81	0.9834		0.37991		

Table 1: Results of the ADONIS analysis on the DPCoA distances. Samples of t0 (before applying any treatments) were excluded for this analysis. Number of permutations: 5000. Time, treatment and their interaction had a significant influence on community composition.

ADONIS separated by treatment:

Fed

	Df	*SS*	*F*	*R2*	*Pr(>F)*
time	1	0.035638	2.5586	0.09284	0.1102
Residuals	25	0.013929		0.90716	

Starved

	Df	*SS*	*F*	*R2*	*Pr(>F)*	
time	1	0.177349	16.199	0.3432	0.0003999	***
Residuals	31	0.010948		0.6568		

Dead

	Df	*SS*	*F*	*R2*	*Pr(>F)*	
time	1	0.058083	4.9093	0.16414	0.005799	**
Residuals	25	0.011831		0.83586		

Individual ADONIS analyses of the treatments. Samples of t0 (before applying any treatments) were excluded for this analysis. Number of permutations: 5000. Time had a significant effect on community composition only in the starving animals and the dead animals but not in the fed animals.
